# Graft selection in arthroscopic anterior cruciate ligament reconstruction

**DOI:** 10.1007/s10195-010-0124-9

**Published:** 2010-12-23

**Authors:** Emilio Romanini, Franca D’Angelo, Salvatore De Masi, Ezio Adriani, Massimiliano Magaletti, Eleonora Lacorte, Paola Laricchiuta, Luciano Sagliocca, Cristina Morciano, Alfonso Mele

**Affiliations:** 1 GLOBE, Gruppo di Lavoro Ortopedia Basata su Prove di Efficacia, Rome, Italy; 2 Artrogruppo, Casa di Cura San Feliciano, Via Val Cannuta 132, 00166 Rome, Italy; 3 Istituto Superiore di Sanità, Rome, Italy; 4 Dipartimento di Prevenzione, Livorno, Italy; 5 Casa di Cura Mater Dei, Rome, Italy; 6 Agenzia Sanitaria Regionale Campania, Naples, Italy

**Keywords:** Anterior cruciate ligament (ACL), Evidence-based guideline, Systematic review, Knee

## Abstract

**Background:**

Anterior cruciate ligament (ACL) surgical reconstruction is performed with the use of an autogenic, allogenic or synthetic graft. The document issued by the Italian National Guidelines System (SNLG, Sistema Nazionale Linee Guida) at the National Institute of Health aims to guide orthopaedic surgeons in selecting the optimal graft for ACL reconstruction using an evidence-based approach.

**Materials and methods:**

A monodisciplinary panel was formed to define a restricted number of clinical questions, develop specific search strategies and critically appraise the literature using the grading of recommendations assessment, development, and evaluation (GRADE) method. The final draft was shared by the panel and then sent to four external referees to assess its readability and clarity, its clinical relevance and the feasibility of recommendations.

**Results:**

Autograft shows moderate superiority compared with allograft, in relation to the relevant outcomes and the quality of selected evidence, after an appropriate risk–benefit assessment. Allograft shows higher failure rate and higher risk of infection. The panel recommends use of autografts; patellar tendon should be the first choice, due to its higher stability, while use of hamstring is indicated for subjects for whom knee pain can represent a particular problem (e.g., some categories of workers).

**Conclusions:**

Autograft shows better performance compared with allograft and no significant heterogeneity in relation to relevant outcomes. The GRADE method allowed collation of all the information needed to draw up the recommendations, and to highlight the core points for discussion.

## Introduction

The anterior cruciate ligament (ACL) plays a crucial role in knee stability, as it contrasts the combined movement of the tibia against the femur, anterior translation and internal rotation. ACL injuries can affect one or both strands (anterior-medial and postero-lateral) and, on the basis of individual characteristics, can affect ligament function and knee stability, raising the need for surgical reconstruction.

Defining the prevalence of this condition is not easy, as lesions are often asymptomatic; a study carried out on a large sample of students from a US college showed that the possibility of ACL injury may be over 3% in 4 years of physical activity, with a higher risk in female population [1].

Surgical reconstruction for primary isolated ACL lesions is performed using autograft (mostly patellar or hamstring tendons) or allograft (allogenic tissue from humans and of different sorts), while use of synthetic ligaments has recently attracted interest after being abandoned in the past due to a high failure rate.

The choice of technique is based on clinical and biomechanical factors, or on tradition and surgeon experience, or for reasons of context, as shown by various investigations carried out among surgeons from different countries [2, 3].

The heterogeneity in the surgical management of this condition raised a need for clarity on the effectiveness of the different types of grafts, through a systematic, critical appraisal of the literature. The National Guidelines System (SNLG, Sistema Nazionale Linee Guida) of the Ministry of Health at the National Institute of Health (Istituto Superiore di Sanità, ISS) therefore engaged in the elaboration of a document aimed at guiding orthopaedic surgeons in the choice of best practice for primary anterior cruciate ligament reconstruction. The document does not evaluate the different systems for graft fixation, nor the different techniques for preoperative preparation or postoperative rehabilitation.

## Materials and methods

The quick review document is an instrument designed to handle very specific clinical issues through a faster process than the one used to draw up guidelines. The panel created to carry out the activities needed to elaborate the quick review is monodisciplinary, in contrast to the one created for guidelines, and aims to answer a small number of clinical questions defined as crucial by the specialists, and to reduce all heterogeneous and sometimes inappropriate clinical practices.

### Panel composition

The panel of experts who collaborated to draw up this document included 14 orthopaedic surgeons, 2 physiatrists, 1 physiotherapist and 2 epidemiologists familiar with evidence-based medicine and the methodology for guidelines development. The working group included representatives from all the main national scientific societies of reference [Gruppo di Lavoro Ortopedia Basata sulle Prove di Efficacia (GLOBE), Società Italiana di Artroscopia (SIA), Società Italiana di Chirurgia del Ginocchio, Artroscopia, Sport, Cartilagine e Tecnologie Ortopediche (SIGASCOT), Società Italiana di Medicina Fisica e Riabilitativa (SIMFER), and Società Italiana di Ortopedia e Traumatologia (SIOT)], supported by a balanced group of independent experts.

All participants signed a declaration of absence of conflict of interests and of acceptance of the methodology as explained during the first meeting.

The panel met twice (4 July 2008 and 6 February 2009), and all materials produced during the process for the elaboration of the document are available at: http://www.snlg-iss.it.

### Definition of the clinical questions, bibliographic search and critical appraisal of literature

The objectives of the document, the clinical questions on the effectiveness and safety of the types of graft to be used for anterior cruciate ligament reconstruction, the inclusion and exclusion criteria for studies and the timeframe to be considered in the bibliographic search were defined by the panel during the first meeting at the Italian National Institute of Health (ISS).

Specific search strategies were defined in accordance with each established clinical question.

The following databases were searched to gather evidence: PubMed, Embase and Cochrane Library, including randomized clinical trials (RCTs) and systematic reviews (SRs) dated 2000–2008.

**Fig. 1:**
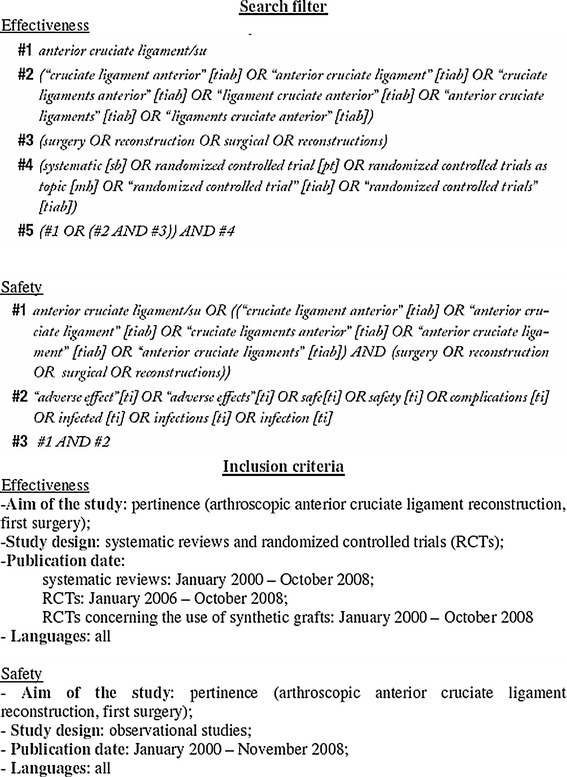
Search strategy and inclusion criteria

Observational studies dated 2000–2008 from the PubMed database were included for questions concerning safety. Figure 1 presents the search filters used for both questions (effectiveness and safety) and the main inclusion criteria.

Qualitative assessment of systematic reviews, RCTs and observational studies was carried out using a structured method [4, 5].

### Data extraction, summary of evidence and recommendations

The selection of studies, their methodological evaluation and the extraction of data were carried out by specifically trained personnel. The evidence gathered from each study was summarized in tables, each specific to a single question and type of study. The summary tables adopted in this document are those defined by the National Institute for Clinical Excellence (NICE), updated in 2007.

The recommendations were drawn up for each clinical question without adopting any specific grading system, that is, without using any structured system to grade the strength of recommendations. The intensity and certainty supporting all recommendations are reported in narrative form, without any symbol, graded score or hierarchy. Each recommendation is introduced by a description of the discussion that led to its definition, to make clear the level of agreement of the working group.

The panel adopted the GRADE system to carry out the critical appraisal of literature and to draw up the recommendations [6–11].

The critical appraisal of the literature was carried out for each outcome considered relevant by the panel, following the principles of this method. The quality of evidence, finally, was related to the assessment of all risks connected to adopting that specific procedure, thus reaching the definition of the recommendation.

### External review

The final draft was shared by the panel in the second and last meeting, and then sent to four external referees, asking them to assess its readability and clarity, its clinical relevance and the feasibility of recommendations. The referee group included renowned orthopaedic surgeons with an interest in knee surgery and with active scientific production in the field. The full text of the document (currently available only in Italian), including all suggestions from the referees, is available on the SNLG website at: http://www.snlg-iss.it.

## Results

The panel agreed on two clinical questions, one related to the effectiveness and the other to the safety of arthroscopic ACL reconstruction carried out using autograft (Table 1), allograft (Table 2) or synthetic graft (Table 3). The literature search gathered 489 titles and abstracts, among which 30 articles met the defined selection criteria.

**Table 1:** Key questions, selected studies and recommendations on use of autograft in arthroscopic ACL reconstruction

Key questions	Studies	Recommendations
Is use of autograft effective in patients with anterior cruciate ligament injury (with or without meniscal lesions and/or grade I/II focal chondral lesions) and a shared indication to arthroscopic reconstruction?	407 identified, 26 selected, 19 rated, 19 included	*Clinical practice* Evidence is currently not sufficient to absolutely recommend use of one of the treated autograft techniques. Higher stability subsequent to use of patellar tendon is proven, while use of hamstring is suggested in patients needing, for various reasons, to stay on their knees for long periods of time, and who therefore need a substantial reduction of intensity and length of pain
Is use of autograft safe in patients with anterior cruciate ligament injury (with or without meniscal lesions and/or grade I/II focal chondral lesions) and a shared indication to arthroscopic reconstruction?	48 identified, 5 selected, 5 rated, 5 included	*Research* The methodological quality of the studies investigating the different autograft techniques is not very high. Randomized studies are therefore needed, with good statistical power, adequate blinding procedures in the choice of outcomes and a standardized definition of interventions and outcomes Qualitative studies are also needed, aimed at investigating patients’ (and clinicians’) preferences in relation to the relevance of the considered outcomes Further studies are finally recommended, aimed at testing the effectiveness of autograft with hamstring associated to extra-articular surgery to contain laxity

**Table 2:** Key questions, selected studies and recommendations on use of allograft in arthroscopic anterior cruciate ligament reconstruction

Key questions	Studies	Recommendations
Is use of allograft effective in patients with anterior cruciate ligament injury (with or without meniscal lesions and/or grade I/II focal chondral lesions) and a shared indication to arthroscopic reconstruction?	407 identified, 3 selected, 2 rated, 2 included	*Clinical practice* Use of autograft is recommended in anterior cruciate ligament reconstruction. Use of allograft shows, in fact, higher failure rate and slightly increased risk of infective complications
Is use of allograft safe in patients with anterior cruciate ligament injury (with or without meniscal lesions and/or grade I/II focal chondral lesions) and a shared indication to arthroscopic reconstruction?	48 identified, 3 selected, 2 rated, 2 included	*Research* Randomized studies are recommended, comparing the best techniques concerning the two types of graft (autograft and allograft) and providing information on the contextual (organizational, structural, cultural) determinants of effectiveness for each intervention

**Table 3:** Key questions, selected studies and recommendations on use of synthetic grafts in arthroscopic anterior cruciate ligament reconstruction

Key questions	Studies	Recommendations
Is use of synthetic grafts effective in patients with anterior cruciate ligament injury (with or without meniscal lesions and/or grade I/II focal chondral lesions) and a shared indication to arthroscopic reconstruction?	235 identified, 3 selected, 2 rated, 2 included	*Clinical practice* Lack of evidence does not allow recommendation of use of synthetic graft for anterior cruciate ligament reconstruction. The little available evidence suggests possible future development of use of such materials, but further studies are needed to assess their effectiveness
Is use of synthetic grafts safe in patients with anterior cruciate ligament injury (with or without meniscal lesions and/or grade I/II focal chondral lesions) and a shared indication to arthroscopic reconstruction?	48 identified, 0 selected, 0 rated, 0 included	*Research* Randomized studies are recommended, aimed at comparing use of synthetic grafts and the best available techniques of autograft and allograft for anterior cruciate ligament reconstruction Studies aimed at identifying synthetic materials and the most appropriate methodologies for their use are also recommended

### Use of autograft in arthroscopic ACL reconstruction

Table 1 reports the question concerning autograft, the literature screening procedure and the recommendations as defined by the panel. Twelve SRs and seven RCTs were selected for the assessment of the effectiveness of autograft, comparing use of patellar tendon (PT) versus hamstring (HS), while five retrospective studies were chosen to define the recommendations concerning safety.

All selected reviews included mostly randomized or quasi-randomized prospective studies based on follow-ups of 2 or more years, and aimed at assessing the effectiveness of autograft using objectively measured or subjectively assessed mechanical or functional outcomes (laxity, stability, return to pre-injury activity and loss of flexibility).

The assessment of laxity and stability defined with various measures [Knee Test (KT), Lachman test, pivot shift test, International Knee Documentation Committee (IKDC) score], the frequency with which patients return to pre-injury activity and the loss of flexibility support the hypothesis that PT in several cases performs better than HS [12–19], while HS appears to reduce anterior pain and loss of extension.

The effect rates reported are often close to statistical significance (even if unable to prove superiority of one specific technique), confirmed by the results of the included RCTs. These [20–25] are not able to demonstrate differences between the two techniques due to the lower statistical power compared with the reviews of primary studies, and often show methodological flaws affecting the inferences.

Promising experiences using four-strand hamstring tendon have also been carried out [26], or using two-strand hamstring tendon associated to extra-articular plastic (2HS EP), to limit laxity in rotation [27].

Both seem to substantially improve HS graft performance in terms of stability, but require further investigation.

Evidence in relation to safety of autograft comes instead from uncontrolled observational studies and refers to infections, and in one case [28] to mechanical and functional side-effects of surgical procedures.

Clusters of joint infections are reported among subjects who underwent ACL reconstruction (1.6–2.6%), with slight predominance with HS use (5.7%) and an increase of risk, probably due to former ACL reconstruction [relative risk (RR) = 5.1] or knee surgery (RR = 1.90) and to the use of some fixation systems for femur (RR = 4.5 for Endobutton) or tibial (RR = 3.2 with metallic post and washers) fractures [29, 30]. The infection rate, in the absence of clusters, results <1%, showing no differences in relation to the technique chosen [31].

Almazàn et al., finally [32], show that donor-site complications are more frequent in HS grafts (6.2% versus 0.6% in PT), as are complications due to complicated procedures.

PT graft appears therefore to be fairly superior to HS graft, in terms of stability, return to pre-injury activity and flexural strength, while use of HS can be reasonably restricted to specific situations, due to its effectiveness in reducing pain and loss of extension. Evidence on safety is scarce and fragmentary, and no inferences can be made apart from a few suggestions on infective complications.

The panel therefore decided to recommend use of PT due to its proven higher stability and to identify at the same time a possible subgroup of subjects for whom knee pain can represent a particular problem (e.g., some categories of workers), or for whom reducing length and intensity of pain as much as possible could be important, and define for this subgroup a specific indication for use of HS.

### Use of allograft in arthroscopic ACL reconstruction

Table 2 summarizes the activities of the panel in relation to use of allograft. Only two SRs were selected at the end of the literature screening. These reviews include non-randomized primary studies aimed at comparing allograft versus autograft.

The study of Prodromos et al. [33] analyzes data from 20 case series from 18 studies on the stability of allograft, comparing them with data from a former meta-analysis on autograft [34].

The global stability rate indicates higher efficacy of autograft, with 72% normal stability (versus 59% registered in the allograft group) and 5.3% abnormal stability (versus 14% registered in the allograft group). The differences observed between the two types of grafts were statistically significant in both cases (*P* < 0.001).

Moreover, higher efficacy of non-irradiated tissues (63%) versus irradiated tissues (43%, *P* < 0.001) has been observed, and of non-patellar tissue (64%) versus patellar tissue (57%, *P* < 0.001).

Krych et al.’s review [35] included one quasi-randomized study and five non-randomized studies comparing effectiveness between autogenic and allogenic patellar tendon graft. The follow-up was longer than 2 years, and the same rehabilitation protocols were adopted.

No statistically significant differences emerged between the two types of grafts apart from the worse performance of allograft in terms of graft failure [odds ratio (OR) = 5.03, 95% confidence interval (CI) 1.38–18.33] and of hop test results <90% versus healthy side (OR = 5.66, 95% CI 3.09–10.36).

The panel, in accordance with the GRADE methodology, was invited to vote on the relevance of the outcomes considered in the selected studies. Table 4 reports the assigned score, the quality score, the estimated effectiveness and the risk–benefit assessment for each outcome.

**Table 4:** Comparison of allograft versus autograft

Outcome	Relevance^a^	Effectiveness rate	Quality of evidence^b^	Risk–benefit
Return to pre-injury activity	8.3-Critical	OR 1.2 (0.7–2.0) *favouring autograft*	+	Slight increase of infective complications in allograft
Graft rupture	8-Critical	OR 5.0 (1.4–18.3) *favouring autograft*	++	
IKDC score	7.7-Critical	OR 1.5 (0.2–10.4) *favouring autograft*	−	The sterilization procedures risk affecting the effectiveness of allograft
Lachman test	5.8-Important	OR 2.7 (0.7–10.8) *favouring autograft*	+	
Pivot shift test	5.8-Important	OR 1.2 (0.5–3.0) *favouring autograft*	+	
Hop test	5-Important	OR 5.7 (3.1–10.4) *favouring autograft*	+	

GRADE method

^a^ 1–3 = unimportant; 4–6 = important; 7–9 = critical

^b^ High = ++++; moderate = +++; low = ++; very low = +

Graft rupture, re-operation rate, return to pre-injury activity and IKDC score were considered critical outcomes, and graft rupture in particular benefited from evidence much higher in quality than that gathered for other outcomes, supporting higher efficacy of autograft versus allograft. The other outcomes defined by the panel seemingly showed higher efficacy of autograft, even if the values did not reach statistical significance.

Evidence in relation to safety, on the other hand, relies on two studies and essentially concerns infective complications.

Centeno et al.’s study [36] was not assessed due to the inadequacy of its design and the irrelevance of the results. Crawford et al.’s study, on the other hand [37], reports a 3.3% (11/331) infection rate among 331 patients who underwent ACL reconstruction between 2000 and 2002. All infections were observed among the 250 patients treated with aseptic allograft (4.4%, 11/250), while no infections were observed among the 81 subjects treated with sterile allograft or autograft. The type of graft (allograft versus autograft, RR = 3.3, n.s.), the type of treatment adopted to process grafts (aseptic versus sterile, RR = 70.5, 95% CI 1.1–∝), use of supplemental tibial staples (use versus non-use, RR = 10, 95% CI 3.0–32.9) and use of a specific device (Intrafix versus no fixation, RR = 10.6, non-significant) resulted as the main risk factors.

Moderate superiority of autograft as ACL reconstruction technique is to be pointed out, on the basis of allograft performance (versus autograft), in relation to the outcomes considered relevant and the quality of selected evidence, subsequent to the risk–benefit assessment. Use of allograft, in fact, shows higher failure rate and higher risk of infective complications with aseptic tissue.

### Use of synthetic grafts in ACL reconstruction

Table 3 reports the question concerning use of synthetic grafts and the scant evidence supporting the recommendations.

The full text of a systematic review identified by the literature search [38] resulted unavailable. The abstract stated that the study included 3 RCTs and 11 case series. Authors concluded that no indications could be stated due to the lack of evidence.

Two more studies (RCTs) gathered by the literature search compared patellar tendon autograft and synthetic graft. The full texts of both of these articles underwent critical appraisal [39, 40], and the studies resulted of good quality, even if based on small populations (40 enrolled in Muren et al.’s study and 53 in Nau et al.’s study).

Muren et al.’s study investigated use of a polypropylene device, the Ligament Augmentation Device (LAD), stitched to the autograft, while Nau et al. investigated the use of the Ligament Advanced Reinforcement System (LARS), a device produced in France, fixed with titanium screws. This trial is included in Pichon Riviere’s systematic review.

Muren et al. follow the 40 randomized patients for 7 years (3 years for arthrometric assessment with KT 1000), reporting no significant differences between the two groups. The results of this study refer to patients with acute ACL injuries, with time of injury less than 3 weeks prior to enrolment being an inclusion criterion.

Nau et al., on the other hand, included patients who suffered ACL injury no less than 6 months before enrolment, and showed a substantial equivalence after arthrometric tests (2.38 mm in PT versus 4.86 mm in LARS, *P* < 0.05) and IKDC assessment. Patients expressed a slight preference [assessed using the Knee injury and Osteoarthritis Outcome Score (KOOS) score] for LARS treatment at 6 and 12 months, but not at 24 months.

No studies were identified investigating the safety of synthetic grafts in ACL reconstruction. The panel agreed on the potential benefits of synthetic grafts in ACL reconstruction; the lack of evidence, however, does not allow recommendation of use of such materials, and further investigations to assess their efficacy and safety are needed.

## Discussion

The document on graft choice in primary ACL surgery is the first SNLG experience of a quick review (*documento di revisione rapida*). The main feature of this type of document, apart from some methodological issues, is the specificity of topics, being mainly monospecialistic.

The GRADE method was used to analyze the allograft question. Its strong structure allowed the collation of all information needed to draw up the recommendation, and highlighted the core points for discussion.

Two reviews [34, 35] underwent critical appraisal in relation to the question concerning allograft. These two reviews included non-randomized studies (except for Gorschewsky’s quasi-randomized study). Kyrch et al.’s review, according to the GRADE method, started from a “low” quality level, while Prodromos et al.’s review, including case series with historic (non-concurrent) controls, started from a “very low” quality level.

The critical appraisal of this evidence raised some difficulties, as the quality resulted often below the “low” or “very low” level. Strongly recommending a specific procedure is embarrassing if the available evidence is of very low quality, even if the method states a certain independence between quality of evidence and strength of recommendation. The undertones needed in shaping recommendations and avoiding such embarrassment did not fit the “weak” and “strong” labels. The panel therefore decided to express recommendation strength in a narrative way, bringing together in the text both structured assessment of evidence and unstructured discussion.

Krych et al.’s study showed some heterogeneity between the included studies. The author stated that this was due to the presence of a single study [41] considering the type of preparation and sterilization used for patellar tissue in allograft. These treatments for sterilization with radiation and dehydration with acetone would have, in other words, decreased the efficacy of allograft, producing data against its use, and caused the heterogeneity of results.

The evaluation of the outcomes, as defined by the GRADE method, enabled verification that the statistical test used by the author highlighted no significant heterogeneity in relation to the outcomes considered relevant (ex. graft failure). The analysis of sensitivity, based on the inclusion/exclusion of Gorschewsky et al.’s study, did not substantially modify the results, causing only a loss of power that did non allow the results to reach statistical relevance. Results, however, showed a certain superiority of autograft.

The panel agreed that the sterilization procedure used in Gorschewsky et al.’s study is to be considered responsible for the lower efficacy of the allograft, but that it is not currently adopted. Therefore, the recommendation defined by the working group did not take into consideration the effects of this procedure on the effectiveness of the intervention.

Global agreement was reached for all questions and recommendations, irrespective of divergences arising in interpretation and assessment of some studies. Evidence, in fact, showed overall homogeneity, and the clinical opinions from each member converged without affecting the richness of information. The results of the review are therefore coherent with current trends in clinical practice, although they do supply robust scientific data to support the choice of graft in ACL primary surgery.

## Conclusions

Available evidence allows recommendation of use of autograft over allograft in arthroscopic ACL reconstruction and to recognize, for autograft, better performance of PT over HS. It is therefore appropriate to select one of these two main choices (PT and HS), assessing the indication on a case-by-case basis. It is also appropriate to consider allograft and artificial ligaments only in very selected cases, discouraging widespread use, given the potential risks and paucity of well-performed, well-designed clinical studies. The indications for further research are also clear. Consolidation of the experience in use of two- and four-strand HS and in using specific techniques to contain laxity is suggested. Further investigations are also strongly suggested on use of synthetic grafts in studies comparing their effectiveness versus autograft. It is valuable to recall that stepwise introduction of new orthopaedic technologies should include preclinical testing, randomized clinical trials, multicenter studies and post-market surveillance, to provide surgeons with adequate information to make informed decisions regarding use of new technologies in their practice, including ACL reconstruction with synthetic ligaments [42].

Finally, this experience confirms the feasibility of practice guidelines to drive an evidence-based approach in orthopaedic surgery. In this particular case, representatives from the scientific societies with an interest in knee surgery (SIOT, SIA, SIGASCOT, SIMFER, and GLOBE) participated in collecting, analyzing and discussing the available data to develop evidence-based guidelines using a standardized and reliable methodology. The practice of evidence-based medicine can be conceptualized as the integration of the best available research evidence, clinical circumstances and patients’ values and preferences. Evidence-based practice guidelines allow practitioners to develop treatments for a specific patient, on the bases of not only his/her experience and personal knowledge, but also the most up-to-date scientific evidence, reviewed and evaluated using a structured, detailed and explicit approach. Through the process of guideline development, clinical and methodological experts evaluate and condense the universe of information available on a clinical issue into a useful set of parameters that the physician can complete with his/her own experience and knowledge in managing a patient. Guidelines are not a substitute for continuing study, rather they represent a tool for the practitioner to provide the best care for his/her patients [43].

## Appendix

Ezio Adriani, Casa di Cura Mater Dei, Roma; Angelo Cacchio, Divisione di Medicina Riabilitativa, Università La Sapienza, Roma; Vincenzo Condello, Ospedale S. Cuore Don Calabria, Negrar (VR); Franca D’Angelo, Istituto Superiore di Sanità; Salvatore De Masi, Dipartimento Prevenzione, ASL 6, Livorno; Bernardino Di Paola, Casa di Cura Mater Dei, Roma; Andrea Foglia, Studi di Fisioterapia Riabilita, Civitanova Marche (MC); Piermaria Fornasari, Istituto Ortopedico Rizzoli, Bologna (Banche del Tessuto Muscolo-scheletrico); Giovanni Giordano, Istituto Ortopedico Rizzoli, Bologna; Roberto Iovine, Azienda U.S.L. di Bologna (Società Italiana di Medicina Fisica e Riabilitativa—SIMFER); Stefano Lupparelli, Università degli Studi, L’Aquila; Massimiliano Magaletti, Artrogruppo, Roma; Maurilio Marcacci, Università degli Studi, Bologna (Società Italiana di Chirurgia del Ginocchio, Artroscopia, Sport, Cartilagine e Tecnologie Ortopediche—SIGASCOT); Giuseppe Milano, Università Cattolica del Sacro Cuore, Roma; Riccardo Minola, Gruppo Policlinico, Monza (Società Italiana di Artroscopia—SIA); Roberto Padua, Ospedale Sandro Pertini, Roma; Giuseppe Rinonapoli, Clinica Ortopedica, Università degli Studi, Perugia; Emilio Romanini, Artrogruppo, Casa di Cura San Feliciano, Rome; Gustavo Zanoli, Casa di Cura S. Maria Maddalena, Rovigo (Gruppo di Lavoro Ortopedia Basata sulle Prove di Efficacia—GLOBE); Claudio Zorzi, Ospedale S. Cuore Don Calabria, Negrar—VR (Società Italiana di Ortopedia e Traumatologia—SIOT).
